# Noninvasive assessment of respiratory muscle strength and activity in Myotonic dystrophy

**DOI:** 10.1371/journal.pone.0177318

**Published:** 2017-06-08

**Authors:** Morgana de Araújo Evangelista, Fernando Augusto Lavezzo Dias, Mário Emílio Teixeira Dourado Júnior, George Carlos do Nascimento, Antonio Sarmento, Lucien Peroni Gualdi, Andrea Aliverti, Vanessa Resqueti, Guilherme Augusto de Freitas Fregonezi

**Affiliations:** 1 PneumoCardioVascular Lab, Departamento de Fisioterapia, Universidade Federal do Rio Grande Do Norte, Natal, Rio Grande do Norte, Brasil; 2 PneumoCardioVascular Lab/HUOL, Empresa Brasileira de Serviços Hospitalares (EBSERH), Universidade Federal do Rio Grande do Norte, Natal, Rio Grande do Norte, Brasil; 3 Departamento de Fisiologia, Universidade Federal do Paraná, Curitiba, Paraná, Brasil; 4 Departamento de Medicina Integrada, Universidade Federal do Rio Grande do Norte, Natal, Rio Grande do Norte, Brasil; 5 Departamento de Engenharia Biomédica, Universidade Federal do Rio Grande do Norte, Natal, Rio Grande do Norte, Brasil; 6 Faculdade de Ciências da Saúde do Trairi, Universidade Federal do Rio Grande do Norte, Santa Cruz, Rio Grande do Norte, Brasil; 7 Dipartimento di Elettronica, Informazione e Bioingegneria, Politecnico di Milano, Milan, Italy; University of Valencia, SPAIN

## Abstract

**Objective:**

To evaluate sensitivity/specificity of the maximum relaxation rate (MRR) of inspiratory muscles, amplitude of electromyographic activity of the sternocleidomastoid (SCM), scalene (SCA), parasternal (2^nd^IS) and rectus abdominis (RA) muscles; lung function and respiratory muscle strength in subjects with Myotonic dystrophy type 1 (DM_1_) compared with healthy subjects.

**Design and methods:**

Quasi-experimental observational study with control group. MRR of inspiratory muscles, lung function and amplitude of the electromyographic activity of SCM, SCA, 2^nd^IS and RA muscles during maximum inspiratory pressure (PImax), maximum expiratory pressure (PEmax) and sniff nasal inspiratory pressure (SNIP) tests were assessed in eighteen DM_1_ subjects and eleven healthy.

**Results:**

MRR was lower in DM_1_ group compared to healthy (P = 0.001) and was considered sensitive and specific to identify disease in DM_1_ and discard it in controls, as well as SNIP% (P = 0.0026), PImax% (P = 0.0077) and PEmax% (P = 0.0002). Contraction time of SCM and SCA was higher in DM_1_ compared to controls, respectively, during PImax (P = 0.023 and P = 0.017) and SNIP (P = 0.015 and P = .0004). The DM_1_ group showed lower PImax (P = .0006), PEmax (P = 0.0002), SNIP (P = 0.0014), and higher electromyographic activity of the SCM (P = 0.002) and SCA (P = 0.004) at rest; of 2^nd^IS (P = 0.003) during PEmax and of SCM (P = 0.02) and SCA (P = 0.03) during SNIP test.

**Conclusions:**

MD_1_ subjects presented restrictive pattern, reduced respiratory muscle strength, muscular electrical activity and MRR when compared to higher compared to controls. In addition, the lower MRR found in MD_1_ subjects showed to be reliable to sensitivity and specificity in identifying the delayed relaxation of respiratory muscles.

## Introduction

Myotonic dystrophy type 1 (MD_1_), or Steinert's disease, is the most common form of dystrophy in adolescents and adults, and the second most common neuromuscular disease [1–3]. The classic form of MD_1_ is characterized by weakness and atrophy in skeletal muscles, myotonia, cardiac conduction abnormalities, cognitive impairment and myocardial changes. Additionally, the involvement of respiratory muscles is common with reduction of respiratory muscles strength, especially expiratory muscles [4–6], resulting in death [4], specifically due to respiratory insufficiency or pneumonia [7],[8].

The respiratory impairment in MD_1_, is rather complex because progressive muscle weakness is associated to different degrees of central respiratory control abnormalities causing alveolar hypoventilation[9]. In fact, CO_2_ insensitivity occur in MD_1_ independently of lung function impairment and respiratory muscle weakness [10]. In addition the participation of myotonia in DM1 respiratory restriction is not fully clarified.

Myotonia is characterized by a delayed relaxation of skeletal muscles after an intense contraction and is one of the most important clinical features of MD_1_ [11]. The presence of myotonia determines the appearance of symptoms such as muscle stiffness and cramps. Although few studies have been published, there are indications of its presence in the respiratory muscles. In 1954, Benaim & Worster-Drougth [12] showed, through electromyography, the presence of myotonia in the intercostal muscles, but not in diaphragm. In 1959, Kilburn et al. [13] observed in two cases a delayed return of the diaphragm to its resting position. Smorto et al. [14] found high amounts of high frequency electrical activity, considered myotonia, in MD_1_ patients. In another case study, Estenne et al. [15] were able to detect abnormal muscle relaxation and registry of myotonia of the diaphragm and abdominal muscle by performing electromyographic and nasal inspiratory pressure test (SNIP) studies in a patient with congenital myotonia (Thompsen disease). However, due to the presence of abnormalities in relaxation of the diaphragm, which was confirmed by variations in abdominal pressure, the presence of myotonia was attributed to the abdominal muscles and not to the diaphragm. In contradiction, Fitting et al. [16] using similar methodology, found no changes in the abdominal muscles, but in the diaphragm. Finally, Rimmer et al. [17] in a study with 11 MD patients, found only two patients with electrical activity consistent to the presence of myotonia, as measured by surface electromyography (sEMG) of the scalene, parasternal and external abdominal oblique muscles. Therefore, the presence of myotonia in the inspiratory muscles of patients with MD is doubtful, since most of the studies involve few patients with MD and, in some cases, inadequate techniques.

For some years, the maximum relaxation rate (MRR) of the inspiratory muscles has been used as an indirect marker of fatigue and overload of the inspiratory muscles. A decreasing MRR of skeletal muscle means muscle overload and precedes the failure of power generation [18, 19]. Considering the muscle relaxation as an active process that consumes energy that can be affected by different conditions, including fatigue, muscle weakness and atrophy of muscle fibers [20], a decreasing MRR during the sniff test could be useful for evaluating the presence of inspiratory muscles overload, signs of myotonia, and fatigue [21–23]. Therefore, the objectives of this study were to evaluate the MRR and electrical activity of the inspiratory muscles in patients with MD_1_. We hypothesized that markers of myotonia could be found in this population by analyzing different parameters describing the strength, the relaxation and the electrical activity of respiratory muscles. More specifically, we have considered maximal inspiratory pressure (PImax), maximal expiratory pressure (PEmax) and SNIP test as indexes of strength and MRR and sEMG as indexes of muscle relaxation and contraction, respectively.

## Methods

### Subjects and study design

This is an observational, quasi-experimental study, with control group [24]. Subjects were individuals diagnosed with MD_1_ recruited from a clinical follow-up with a neurologist at a University Hospital from January 2015 to June 2016. All patients and healthy subjects were assessed in a single day after receiving initial information and recommendations on the preparation for the assessments.

Inclusion criteria for the MD_1_ group were: patients aged between 18 and 65 years, properly diagnosed and accompanied by a neurologist. Those who were smokers (or ex-smokers) or presented obstructive respiratory disease, musculoskeletal comorbidities or impaired intellectual ability (established by medical diagnosis) that would prevent the assessments, who failed to perform tests for other reasons or quit from the study were excluded. The control group included self-reported healthy subjects matched for gender and age. Individuals who were smokers (or ex-smokers) or presented cardiac, respiratory or musculoskeletal comorbidities, deviated septum, rhinitis or nasal obstruction, as well as those who failed to perform the tests or gave up from participating of study were excluded. Additionally, healthy subjects should not use any type of drug that could interfere with the test results, such as antihypertensive or bronchodilator drugs. All subjects agreed to participate in the study by signing an informed consent form, which was approved by the Ethics Committee of the Federal University of Rio Grande do Norte/Brazil (protocol n^o^: 752.322) according to the Declaration of Helsinki.

### Clinical phenotype and muscle impairment MD_1_ classification

The MD_1_ patients were classified in relation to degree of muscle impairment and clinical phenotypes [25] by the neurologist according to the Muscle Impairment Rating Scale (MIRS) [26], and the MD_1_-specific form according to age and onset and clinical symptoms. The MIRS is structure in five degrees of impairment in relation to muscle involvement, progressing from distal to proximal: grade 1, no muscular impairment; grade 2, minimal signs (myotonia and digit flexors and craneal muscle wasting and weakness); grade 3, distal weakness (no proximal weakness except isolated elbow extensor weakness); grade 4, mild to moderate proximal weakness; grade 5, severe proximal weakness. The phenotype classification includes four categories: 1- congenital, 2- juvenile, 3- classic adult onset and 4- mild late-onset.

### Lung function

Spirometry was performed through a KoKo DigiDoser^®^ spirometer (nSpire Health, Inc. Longmont, USA) and considered complete when three acceptable curves were produced (with variation equal to or lower than 5% of the highest value). The technical procedure, the acceptance and reproducibility criteria as well as the standardization of equipment followed the recommendations of the ATS/ERS [27]. The reference values used in this study had been previously published for the Brazilian population [28].

### Respiratory muscle strength

Respiratory muscle strength was assessed from measurements of PImax, PEmax and SNIP by a digital manometer (NEPEB-LabCare/UFMG, Belo Horizonte, Brazil), following the procedures previously published by the Brazilian Society of Pneumology [29]. PImax was measured from residual volume and PEmax from total lung capacity, using a nose clip. The highest value obtained in a maximum of five tests (with variation equal to or lower than 10% of the highest value) was used for each assessment. SNIP was obtained from functional residual capacity, with the subject having one nasal cavity occluded by a plug coupled to a catheter connected to a digital manometer, while maintaining the other nostril open[30]. Previously published reference values were used [31].

In order to identify respiratory muscle weakness, cutoff values were adopted and calculated as the mean of the normal population, published previously, minus 1.96 multiplied by the standard deviation. The reference values of Neder et al. [31] were used for PImax and PEmax; and the reference values of Araujo et al. [30] were used for SNIP. The cutoff values established for men and women, respectively, were: 59.1 cmH_2_O and 70.4 cmH_2_O for PImax; 58.2 cmH_2_O and 53.9 cmH_2_O for SNIP; 101.1 cmH_2_O and 69.2 cmH_2_O for PEmax.

### Assessment of maximum relaxation rate of inspiratory muscles

MRR was derived from SNIP test and calculated using LabChart Reader 8.0 software (ADInstruments, New Zealand). The values were obtained as the first derivative of pressure-time curve (dP/dT) over the first half of the relaxation curve by calculating the tangent at the steepest part of the pressure curve. To exclude the effect of pressure oscillation amplitude on MRR [21], dP/dT was normalized and expressed as percentage of the pressure drop in every 10 milliseconds (dP/dt) / Psniff*100 (%10ms) [22]. The following criteria were established for choosing the best SNIP value for calculation of MRR: (1) sniff maneuver performed from FRC; (2) peak pressure maintained for less than 50 milliseconds; (3) total duration of the maneuver less than 500 milliseconds; and (4) shape of SNIP curve showing soft peaked curves with upward and downward movement [21],[22].

### Surface electromyography

Capture and processing of myoelectric signals were performed using TeleMyo DTS Desk Receiver^®^ (Noraxon, USA, Inc., Scottsdale, USA) electromyograph and 4 wireless Clinical DTS (Noraxon, USA, Inc., USA) sensors with 16-bit resolution and common-mode rejection ratio > 100 dB. The sampling frequency of the captured signals was 1500 Hz, with 500 Hz low pass filter and signals pre-amplified 1000 times. The software used to capture and store the myoelectric signals during PImax, PEmax and SNIP tests was MR 3.2 (Noraxon, USA, Inc., USA). Ag/AgCl bipolar surface electrodes were placed along the direction of the muscle fibers, all in the right side of the body to avoid contamination by the cardiac electrical signals. The skin of the volunteers was properly prepared in order to reduce impedance, favoring the capture of a signal with greater amplitude, with less interference and noise [32]. To capture electromyographic signals, all recommended procedures were strictly followed [33].

Electrodes were placed over the parasternal muscle on the second intercostal space (2^nd^IS), 3 cm from the sternum; the sternocleidomastoid muscle (SCM), on the muscle belly, 5 cm from the mastoid process; the rectus abdominis (RA), 4 cm from the umbilical scar; and the scalene muscle (SCA), on the muscle belly, 5 cm from the sternoclavicular joint and 2 cm above that mark [34]. Electromyographic signals were analyzed using the RMS (root mean square) and normalized from the average of three maximum voluntary isometric contractions for SCM, SCA, RA [35]; and three SNIP and PImax tests for 2^nd^IS [36].

In order to quantify possible delayed muscle relaxation, the duration of electrical activity of each muscle studied was measured and normalized by the duration of the different maneuvers (SNIP, PImax and PEmax). The duration of each respiratory muscle contraction (i.e., the difference between onset and offset time during the maneuver) was automatically calculated by analyzing the time course of pressure curves using the MR3.2 software (MyoMuscle Module, Noraxon, USA).

### Sample size and statistical analysis

Sample size was calculated through a pilot study with five MD_1_ subjects. The standard deviation of the MRR variable was used for a hypothetical t test. Twelve subjects in each group were required to achieve 80% power with P<0.05. Data normality were checked using Shapiro-Wilk test . Parametric data were compared using the Unpaired t-test, while non-parametric data were compared using the Mann-Whitney test. Significant levels were set at a 2-sided P<0.05.

Receiver operating characteristic curves (ROC) were calculated using the MRR, SNIP%, PImax%, PEmax% between MD_1_ patients and healthy subjects. ROC curve was calculated as described previously by Hanley and McNeil [37]. Prism^®^ version 5 (GraphPad Software Inc, USA) was used for data analysis and G*Power version 3.1.9.2 (Franz Faul—Universität Kiel, Germany) for sample size, effect-size and power calculation.

## Results

### Sample size

**Fig 1:**
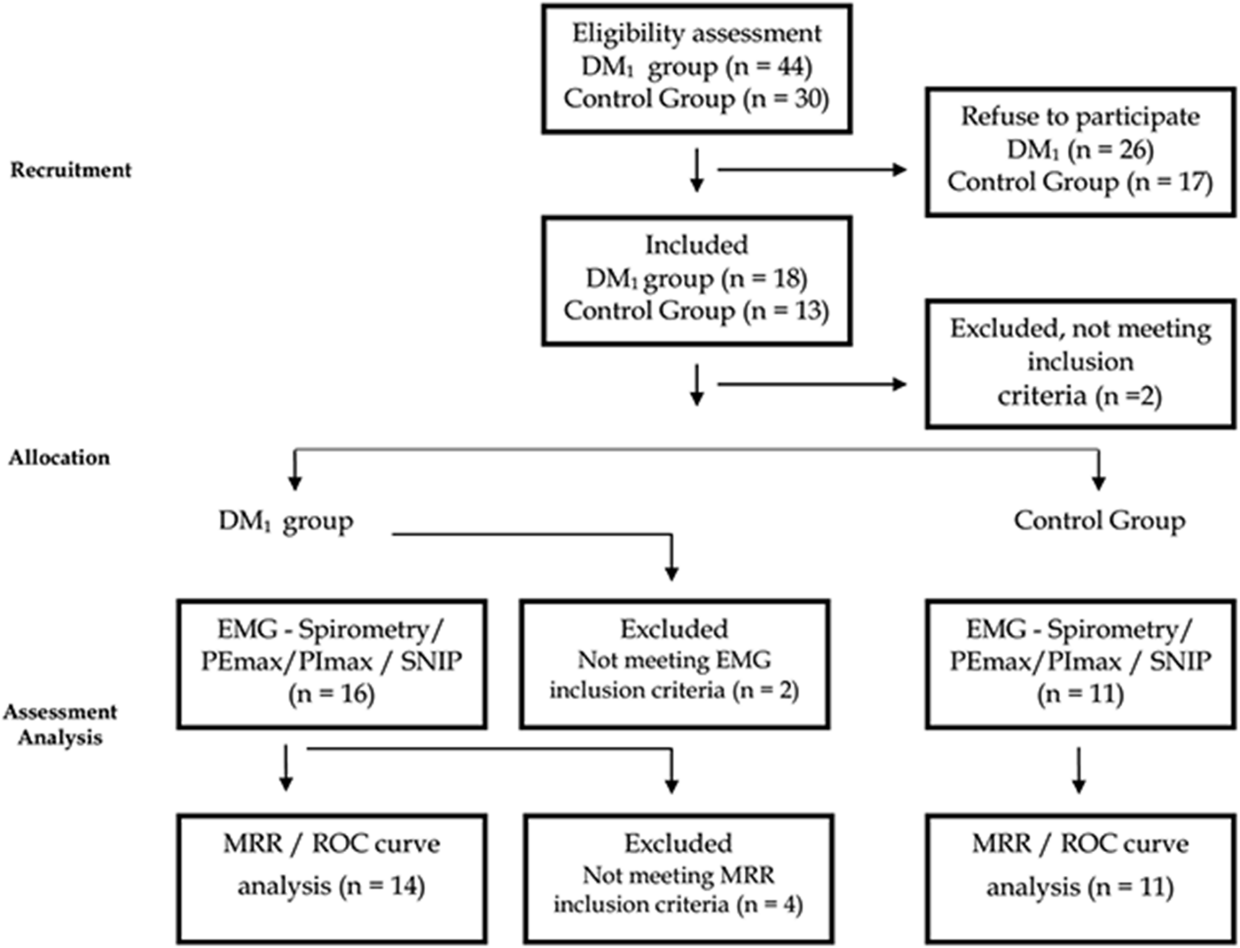
Study flowchart.

Initially, 74 individuals were invited to participate in the study: 44 patients with MD_1_ and 30 healthy subjects. After exclusion criteria a total of 18 MD_1_ subjects and 11 healthy were included in the study (Fig 1).

Considering the mean of each group and the difference between standard deviation of MRR variable, a Cohen’s d = 3.89 (considered large) [38] was found. The power of the study (1-ß), considered the effect size found, was 1.0.

### Symptoms, severity and rate of progression of the disease

Demographic features of the patients are reported in Table 1. The mean age of the patients at time of evaluation was 42.3 ± 11.3 and the mean time elapsed from diagnosis were 6.83 ± 5.98 years. In relation to phenotype, 88.8% (n = 16) were classified as a Classic form and 11.2% (n = 2) as a Mild type. Regarding the classification on MIRS we found that 11,11% (n = 2) were classified as MIRS 1, 55.5% (n = 10) classified as MIRS 2, 22.22% (n = 4) classified as MIRS 3 and 11,11% (n = 2) classified as MIRS 4.

**Table 1:** Anthropometric data, lung function, respiratory muscle strength and maximum relaxation rate of inspiratory muscles.

Variable	Control Group	DM_1_ Group	*P* value
**Gender F/M**	4/7	7/11	-
**Age (years)**	37.4 ± 10.7	42.3 ± 11.3	.2743
**BMI (Kg/m**^**2**^**)**	25.5 ± 4.2	24.9 ± 3.6	.7231
**FVC (%pred.)**	92.6 ± 11.9	72.1 ± 16.6	.0018
**FEV**_**1**_ **(%pred.)**	93.1 ± 10.9	70.2 ± 16.5	.0004
**FEV**_**1**_**/FVC (%pred.)**	101 ± 4.1	97.4 ± 5.6	.0781
**PImax (cmH**_**2**_**O)**	105 ± 29.1	62.6 ± 26.1	.0006
**PImax%**	99 (IQR: 75.3–115)	48 (IRQ: 37–57)	.0016
**PEmax (cmH**_**2**_**O)**	114 (IQR: 101–145)	43.5 (IQR: 36.2–62.7)	.0002
**PEmax%**	102 (IRQ: 89–132.6)	39.7 (IRQ: 28.5–60.5)	< .001
**SNIP (cmH**_**2**_**O)**	72 (IQR: 63–89)	46 (IQR: 36,2–60,5)	.0014
**SNIP%**	70.64 ± 19.6	46 ± 16.26	.0015
***MRR*%/10ms**	7.9 ± 1.7	5.7 ± 1.2	.0010

Data presented as mean and standard deviation (parametric variables—Unpaired t test) and median and interquartile range (nonparametric variables—Mann-Whitney test). BMI—Body Mass Index; Kg/m^2^—kilograms per square meter; FVC—forced vital capacity; % pred.- percentage of the value compared to the predicted value; FEV_1_—forced expiratory volume in one second; PImax—maximum inspiratory pressure; cmH_2_O - centimeters of water; PEmax—maximal expiratory pressure; IQR—interquartile range; SNIP—sniff nasal inspiratory pressure; MRR—maximum relaxation rate. The MRR analysis was conducted with 14 patients.

### Pulmonary function and respiratory muscle strength

The spirometric values for the MD_1_ group showed, on average, a moderate restrictive pattern [39]. Regarding inspiratory muscle strength in the control group, 25% of women were below the cutoff point, while in the MD_1_ group was 40%. Regarding male gender, 14.3% of control group and 54.5% of MD_1_ group were below the cutoff point. In the SNIP test, all subjects were above the cutoff point in the control group, while in the MD_1_ group 63.6% of men and 80% of women were below that point. In the PEmax assessment, where the biggest difference was observed between the two groups, all healthy subjects met or were above the cutoff point for muscle weakness. In the MD_1_ group, 90.9% of men and 80% of women did not reach that value.

**Fig 2:**
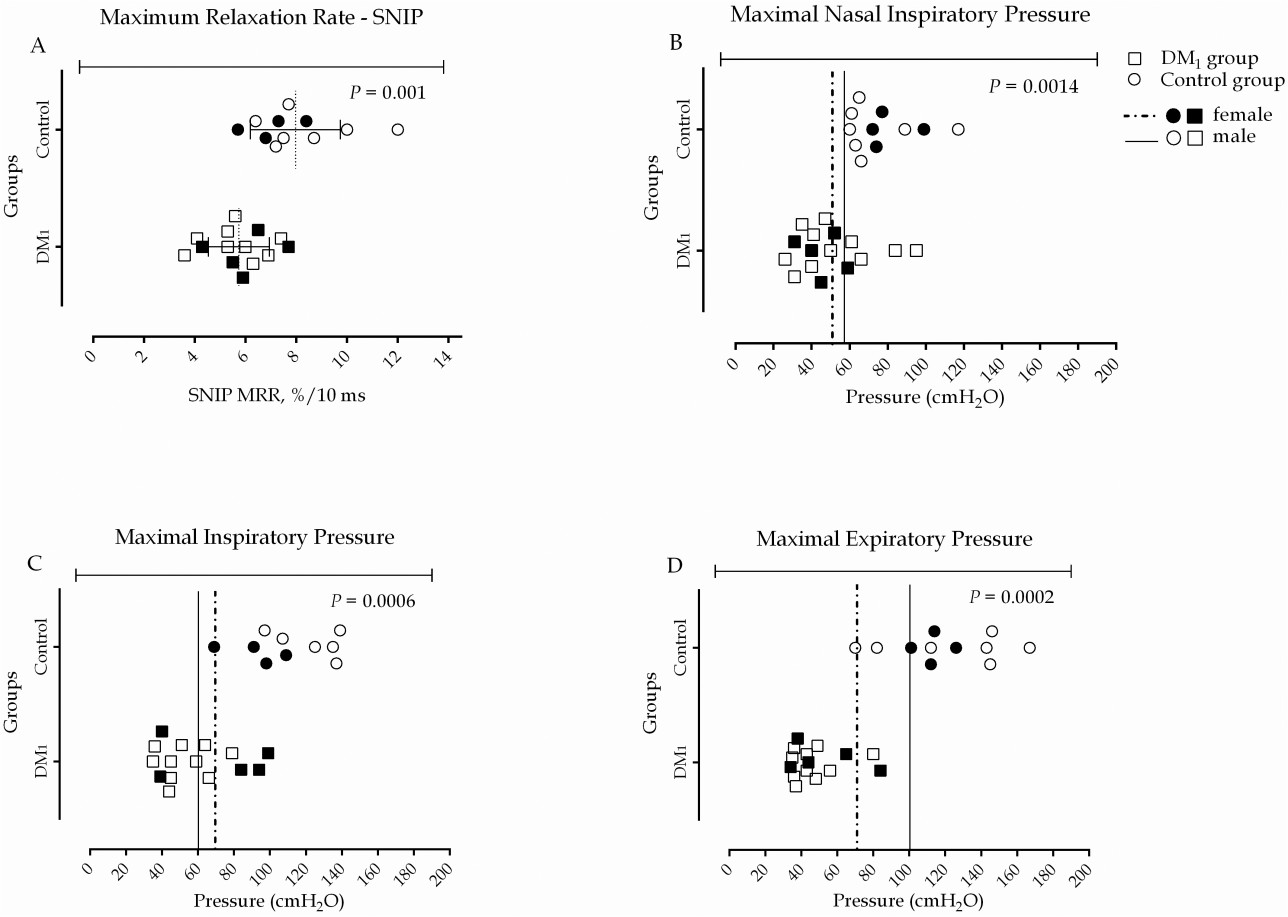
Comparison between MRR (A), SNIP (B), PImax (C) and PEmax (D) for DM_1_ and control group. Data and cutoffs of respiratory muscle weakness for PImax, PEmax and SNIP are presented in absolute values. The cutoff points established for male and female, respectively, were: 59.1 cmH_2_O and 70.4 cmH_2_O, for PImax; 58.2 cmH_2_O and 53.9 cmH_2_O, for SNIP; 101.1 cmH_2_O and 69.2 cmH_2_O for PEmax. All data showed parametric distribution and were compared using Unpaired t test. Differences were considered significant when P<0.05.

For all maneuvers previously mentioned, the differences between the two groups were statistically significant: in absolute values SNIP (P = 0.0014), PImax (P = 0.0006), PEmax (P = 0.0002) and in percentage of predict values SNIP (P = 0.0015), PImax (P = 0.0016) and PEmax (P<0.0001). Spirometric and respiratory muscle strength data are shown in Table 1 and Fig 2.

### Electromyography of the respiratory muscles

**Fig 3:**
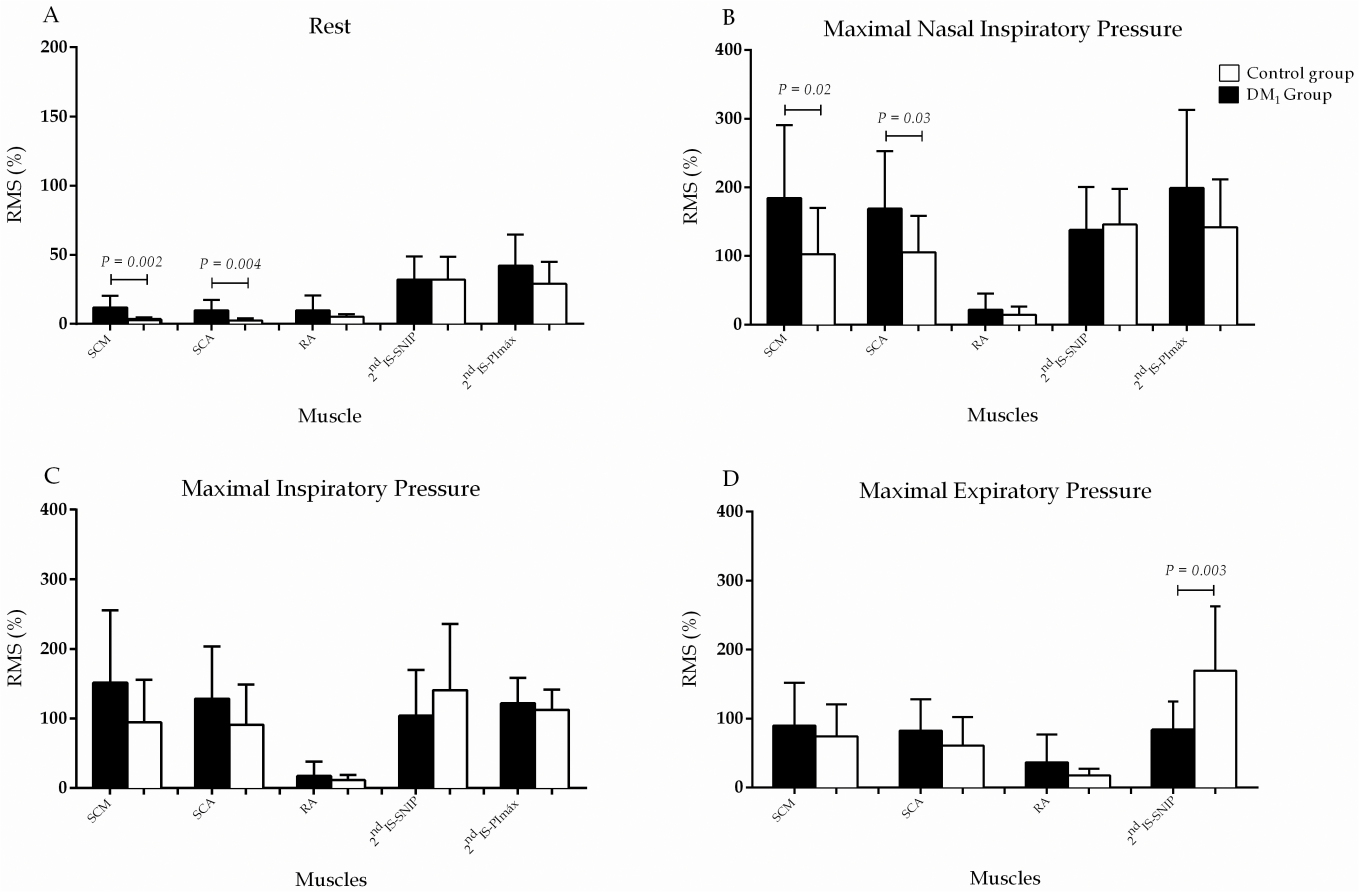
Surface electromyography during the electrical activity of the muscles SCM, SCA, RA and 2^nd^IS at rest (A), SNIP (B), PImax (C) and PEmax (D). Values were normalized and are shown in normalized data (% of RMS at rest) in SCM, SCA and RA. The normalized data from 2^nd^IS was done considering the RMS of maximal values of SNIP and PImax maneuvers of each muscle, during the maneuvers. The sEMG measure at 2^nd^IS was normalized by SNIP and PImax, both maximal measures of inspiratory muscles. All data presented non-parametric distribution and were compared using the Mann-Whitney test. Differences were considered significant when P<0.05.

In the MD_1_ group, 2 subjects were excluded due to the poor quality of the signal captured by the sEMG. Obtained signals from the surface electromyography showed increased muscle activity in MD_1_ subjects in most of the maneuvers carried out, with significant differences found at rest for the SCM (P = 0.002) and SCA (P = 0.004). It was also found significant difference during the PEmax test for the 2^nd^IS (P = 0.003), and during SNIP for the SCM (P = 0.02) and SCA (P = 0.03) (Fig 3).

**Fig 4:**
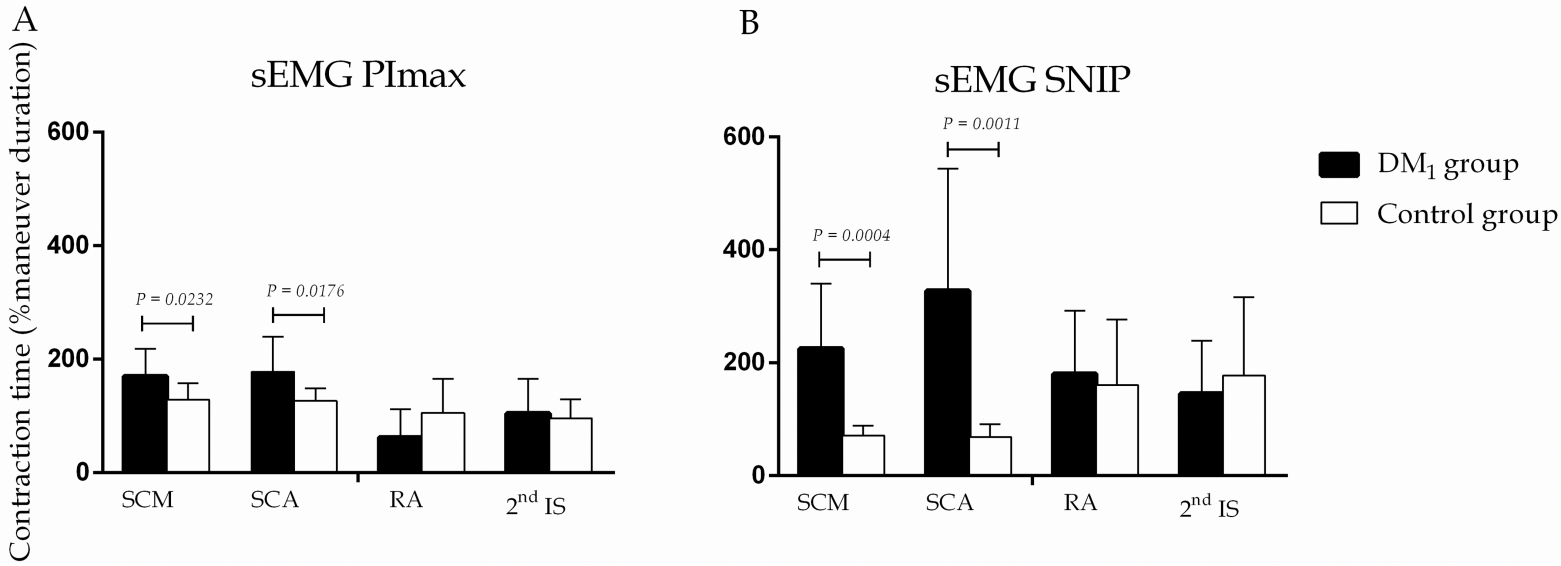
Time of electrical activity of SCM, SCA, RA and 2^nd^IS muscles during SNIP (A) and PImax (B) assessment. Values are expressed in seconds. All data showed parametric distribution and were compared using the unpaired t-test with Welch’s correction. Differences were considered significant when P<0.05.

Electrical activity time of respiratory muscles in patients with DM_1_ was significantly increased compared to controls (Fig 4).

### Maximum relaxation rate of inspiratory muscles

To assess MRR, four DM_1_ subjects were excluded from the analysis because their SNIP test did not meet the criteria for proper data acquisition. The MRR was significant lower in MD_1_ group compared to control group (P = 0.001; 5.7±1.2 vs. 7.9±1.7, respectively) (Fig 2).

### ROC analysis

**Fig 5:**
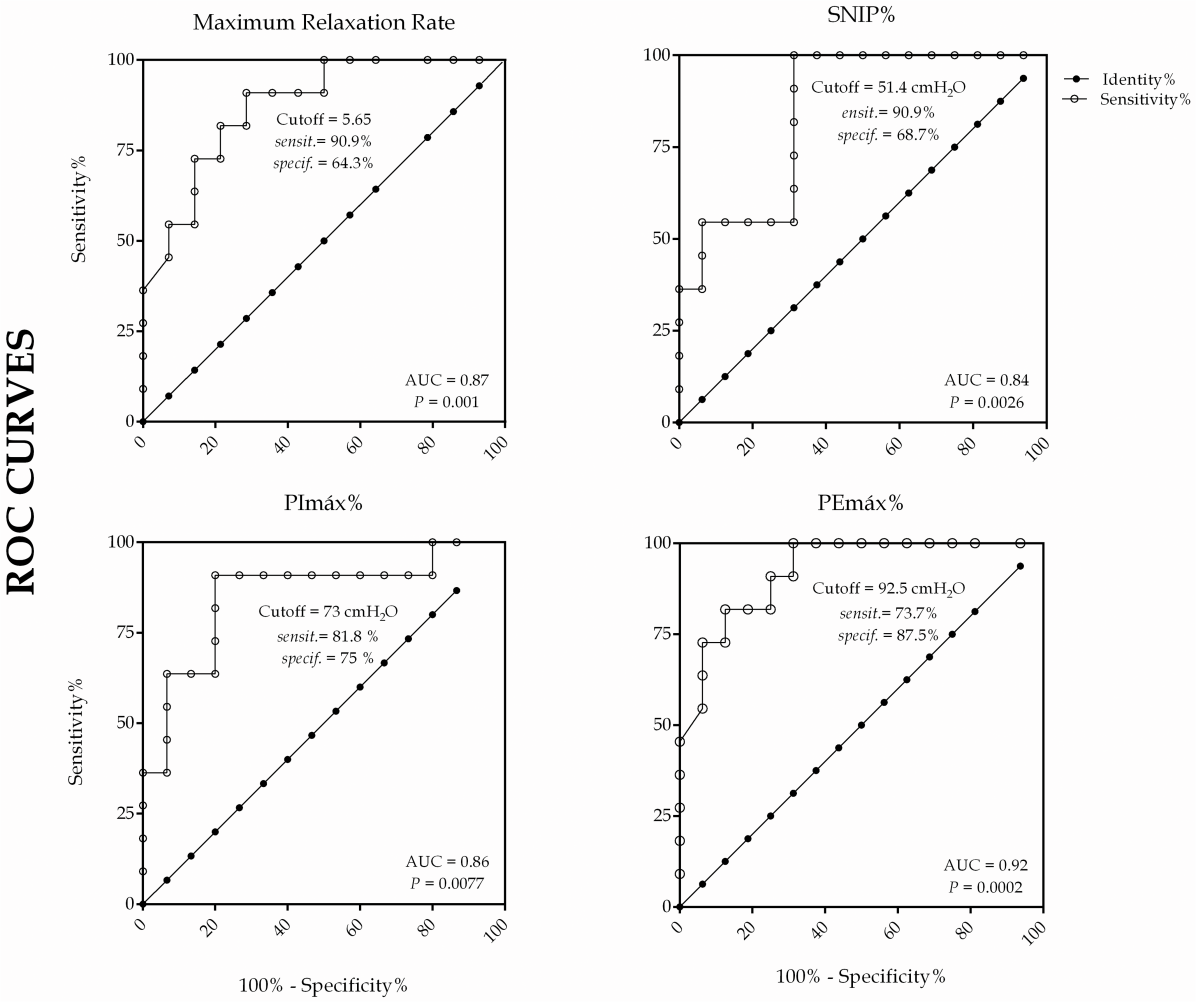
ROC curves of MRR, % predicted of SNIP, PImax e PEmax for DM_1_ and control group.

The results, considering the Area Under the Curve, showed, for all variables, a good ability to discriminate between those individuals with the disease and those without it. The thresholds of sensitivity/specificity, confidential interval of area and p values are shown in Fig 5.

The area under the curve (AUC) above 0.80 associated with a P<0.05, for all the variables (maneuvers), lead to the conclusion that these variables show a good ability to discriminate between those individuals with the disease and those without it.

## Discussion

The study results showed that the MRR is reduced in MD_1_ subjects compared to healthy. The MRR of inspiratory muscles was considered sensitive to identify the presence of reduced MRR in MD_1_ patients and specific to discard it in healthy individuals. According to the results of the ROC curve analysis, which determined a cutoff point of 5.65; the electrical activity of the accessory muscles of respiration (SCM, SCA and RA) was increased in MD_1_ compared to controls (P = 0.004; P = 0.009; P = 0.045, respectively). In addition, lung function was also altered in MD_1_ group as well as reduced respiratory muscle strength showing considered as muscle weakness.

The MRR of inspiratory muscles has been studied for about 25 years. This is an index of fatigue and overload of inspiratory muscles, developed from sniff maneuvers obtained by measuring esophageal or transdiaphragmatic pressure, which are invasive methods [19,20,22]. Subsequent studies [21,36] have observed a high relationship between the MRR obtained through sniff maneuvers associated with esophageal pressure and with nasal pressure, providing a non-invasive measurement of that variable. Despite the technical characteristics of MRR as a measure capable of identifying the delay in inspiratory muscle relaxation in MD_1_ and, therefore, suitable for assessing the presence of myotonia, we have not found previous studies of MRR obtained through nasal inspiratory pressure in MD_1_.

Fitting & Leuenberger [16] were the first authors to study MRR in a single case in 1989. These authors evaluated the effects of procainamide, an anti-myotonic drug, in the diaphragm through transdiaphragmatic pressure. Due to the type of study and the technique used, comparisons are inappropriate. Another study published by Jammes et al. [40], aimed to analyze the electrical activity of the diaphragm and intercostal muscle during loaded inspiratory and expiratory breathing. The authors suggest that the absence of relaxation in diaphragm muscle during expedition may be related to the persistence of repetitive discharge due to a failure o neural inhibitory circuits or to myotonia. More recently, Garcia-Rio et al [41] were the first to assess noninvasively the MRR of inspiratory muscles using SNIP curve in 20 patients with different neuromuscular diseases (5 amyotrophic lateral sclerosis, 3 duchenne muscular dystrophy, 2 myotonic dystrophy, 7 myasthenia gravis, 2 congenital myopathy and brachial plexus palsy) and 10 healthy subjects. These authors used a similar methodology to calculate MRR and found a significantly lower MRR in the neuromuscular group compared to healthy (7.21 ± 0.68 vs. 9.31 ± 1.22). Regarding our results, we also found a significantly lower MRR in MD_1_ patients however; the study of Garcia-Rio et al. used a heterogeneous sample with several different neuromuscular diseases.

Neuromuscular disorders, despite having some common physiopathological characteristics, have varying degrees of severity and progression, which makes unsuitable grouping these diseases for assessment of MRR and its subsequent comparison to a control group. It is important to report that the MD_1_ is characterized by the presence of myotonia in the skeletal muscles and possibly, in the inspiratory muscles, which could potentially affect the relaxation of these muscles and reduce the MRR. Regarding the ROC curve, the high sensitivity and specificity found for MRR quantifies with high statistical value the overall capacity of the MRR to discriminate the pattern of inspiratory muscle relaxation in MD_1_ compared with healthy subjects. Regarding pulmonary function, our study confirmed previous results [42] as it was found the presence of moderate restrictive disorder in MD_1_. In relation to respiratory muscle strength, Gillam et al [4] presented, in a series of 10 patients, values considered below the cutoff point for muscle weakness of the expiratory muscles in 50% of subjects, with values less than 80 cmH_2_O for men and 60 cmH_2_O for women. Recently, similar results have been reported in the literature, in which approximately 50% of patients presented expiratory muscle strength with values below the cutoff point for expiratory muscle weakness [6]. These same authors also showed PEmax, PImax and SNIP percentage of predicted values of about 64%, 70% and 80%, respectively. Additionally, in a study analyzing respiratory muscle strength involving different neuromuscular diseases, patients with MD_1_ have shown a reduction in respiratory muscle strength and PEmax/PImax ratio below normal [5]. In the present study, changes in respiratory muscles strength were reinforced by the changes observed in the sEMG.

A major limitation of our study was the small sample size due to the limited time and financial support. However, we may consider the high power and effect size found and the low prevalence of the disease. Additionally, the results found in our study add new perspectives regarding the MRR assessment (through the SNIP test) of subjects with MD_1_. We must interpret the results of non-invasive MRR of inspiratory muscles in MD_1_ with caution, considering that non-invasive MRR of inspiratory muscles in MD_1_ should be validated with golden standard methods as an invasive measurements of MRR during assessment of SNIP.

## Conclusion

The study results may provide information that can contribute to a better understanding of myotonic dystrophy type 1 and its impact on the respiratory system (either in its functional or muscular aspect). These results may also contribute to the development of new evaluation methods that may be routinely used in clinical practice. Moreover, we have brought new approaches to the surface electromyography, which is still underused to assess respiratory muscles and may be a complementary method for neuromuscular diseases researches.

## Abbreviations

2^nd^IS: Parasternal muscle on the second intercostal space
MD_1_: Myotonic dystrophy type 1
MRR: Maximum relaxation rate
PEmax: Maximum expiratory pressure
PImax: Maximum inspiratory pressure
RA: Rectus abdominis
SCA: Scalene
SCM: Sternocleidomastoid
sEMG: Surface electromyography
SNIP: Sniff nasal inspiratory pressure
